# *trans*-Tris(4-bromo­phen­yl)dichlorido­antimony(V)

**DOI:** 10.1107/S160053681204809X

**Published:** 2012-11-28

**Authors:** Yanling Qiao, Jin Jiang, Jichun Cui

**Affiliations:** a College of Chemistry and Chemical Engineering, Liaocheng University, Shandong 252059, People’s Republic of China

## Abstract

The Sb^V^ atom in the title compound, [SbCl_2_(C_6_H_4_Br)_3_], has an almost regular trigonal–bipyramidal geometry with the equatorial plane made up of three C atoms of the bromo­phenyl groups and the axial positions occupied by two Cl^−^ ions in a *trans* configuration. In the crystal, C—H⋯Br hydrogen bonds link the mol­ecules into zigzag chains along the *b*-axis direction. Pairs of C—H⋯Cl hydrogen bonds further link mol­ecules into cyclic dimers with *R*_2_^2^(10) ring motifs, generating a three-dimensional network.

## Related literature

For related structures, see: Mahalakshmi *et al.* (2001 ▶); Sharutin *et al.* (2010 ▶). For hydrogen-bond motifs, see: Bernstein *et al.* (1995 ▶).
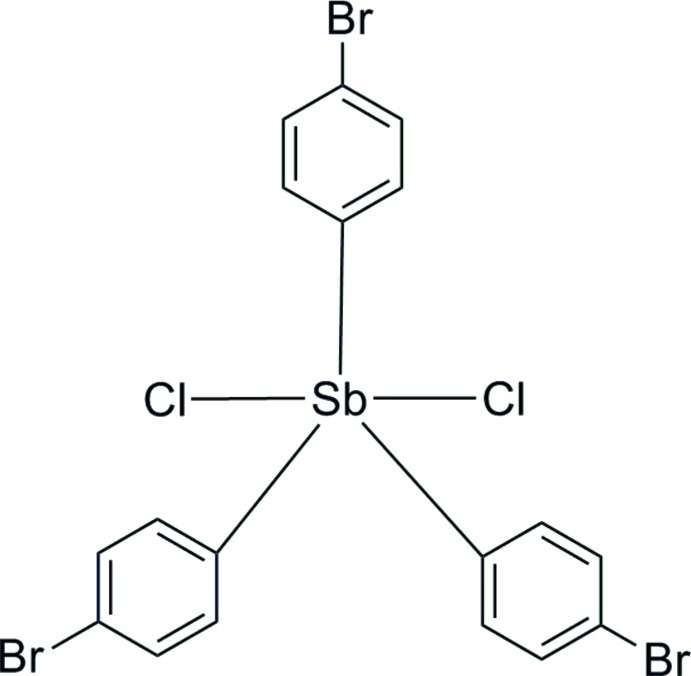


## Experimental

### 

#### Crystal data

[SbCl_2_(C_6_H_4_Br)_3_]
*M* *_r_* = 660.66
Monoclinic, 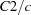

*a* = 15.1050 (13) Å
*b* = 20.124 (2) Å
*c* = 15.1701 (14) Å
β = 117.748 (1)°
*V* = 4081.0 (7) Å^3^
*Z* = 8
Mo *K*α radiation
μ = 7.49 mm^−1^
*T* = 298 K
0.26 × 0.22 × 0.12 mm

#### Data collection

Bruker SMART CCD area-detector diffractometer
Absorption correction: multi-scan (*SADABS*; Sheldrick, 1996 ▶) *T*_min_ = 0.246, *T*_max_ = 0.467
10608 measured reflections
3582 independent reflections
2159 reflections with *I* > 2σ(*I*)
*R*_int_ = 0.060

#### Refinement

*R*[*F* ^2^ > 2σ(*F* ^2^)] = 0.040
*wR*(*F* ^2^) = 0.097
*S* = 0.91
3582 reflections
217 parameters
H-atom parameters constrained
Δρ_max_ = 0.90 e Å^−3^
Δρ_min_ = −0.61 e Å^−3^

### 

Data collection: *SMART* (Bruker, 2007 ▶); cell refinement: *SAINT* (Bruker, 2007 ▶); data reduction: *SAINT*; program(s) used to solve structure: *SHELXS97* (Sheldrick, 2008 ▶); program(s) used to refine structure: *SHELXL97* (Sheldrick, 2008 ▶); molecular graphics: *SHELXTL* (Sheldrick, 2008 ▶) and *Mercury* (Macrae *et al.*, 2008 ▶); software used to prepare material for publication: *SHELXTL*.

## Supplementary Material

Crystal structure: contains datablock(s) I, global. DOI: 10.1107/S160053681204809X/sj5279sup1.cif

Structure factors: contains datablock(s) I. DOI: 10.1107/S160053681204809X/sj5279Isup2.hkl

Additional supplementary materials:  crystallographic information; 3D view; checkCIF report

## Figures and Tables

**Table 1:** Selected bond lengths (Å)

Sb1—C1	2.129 (6)
Sb1—C7	2.119 (6)
Sb1—C13	2.132 (7)
Sb1—Cl1	2.4566 (16)
Sb1—Cl2	2.4716 (17)

**Table 2:** Hydrogen-bond geometry (Å, °)

*D*—H⋯*A*	*D*—H	H⋯*A*	*D*⋯*A*	*D*—H⋯*A*
C2—H2⋯Cl2^i^	0.93	2.93	3.723 (7)	144
C17—H17⋯Br1^ii^	0.93	2.99	3.900 (9)	167

Symmetry codes: (i) 
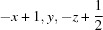
; (ii) 
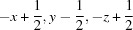
.

**Fig. 1.**
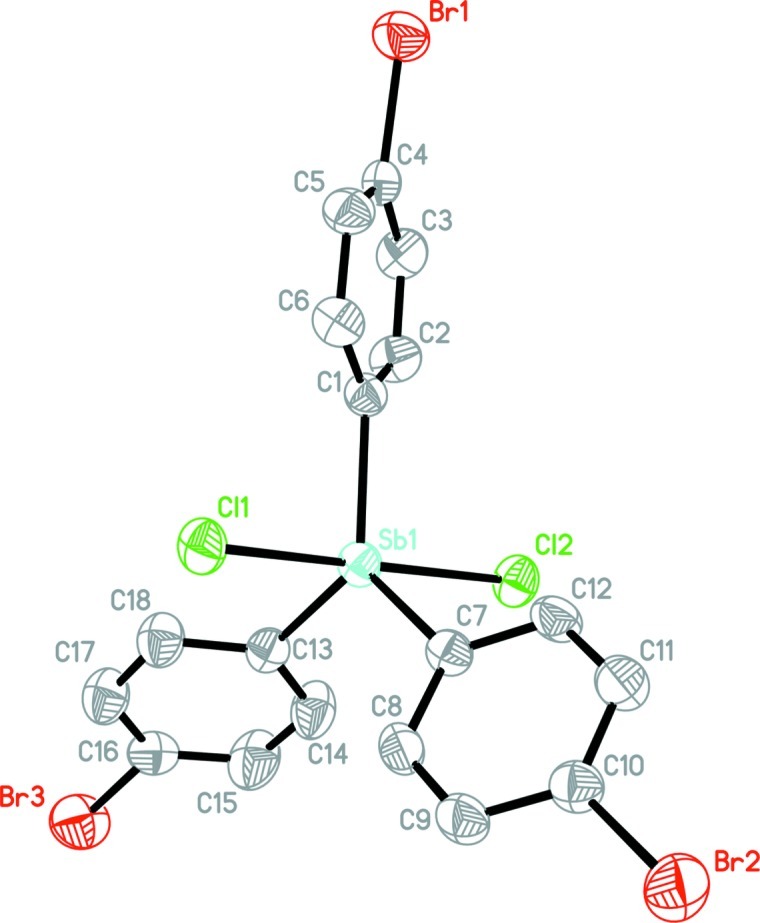
The molecular structure of the compound, showing 50% probability displacement ellipsoids. H atoms have been omitted for clarity.

**Fig. 2.**
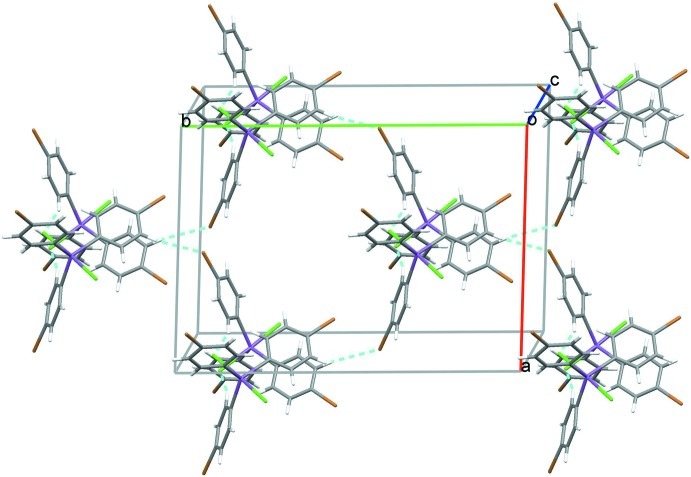
Packing diagram.

## supplementary crystallographic information

### Comment

The molecular structure of the compound is depicted in Fig.1. The Sb atom has an almost regular trigonal bipyramid geometry with the equatorial plane made up of three C atoms, C1, C7 and C13 of the bromophenyl ligands and the axial positions occupied by two Cl^-^ ions. C17—H17···Br1 hydrogen bonds link the molecules into zig-zag chains along *b*. Pairs of C2—H2···Cl2 hydrogen bonds further link molecules into cyclic dimers with R^2^2_2_(10) ring motifs (Bernstein *et al.*, 1995) generating a three-dimensional network.

### Experimental

All operations were carried out under a protective nitrogen atmosphere. 1,4-dibromobenzene (0.5 mol), dissolved in dry ether was added dropwise to Mg turnings (0.5 mol) at 0 °C. The resulting Grignard solution was cooled to -12 °C and antimony(III)chloride (0.1 mol) dissolved in dry ether was added. This mixture was stirred for an additional hour at room temperature and after completion of the reaction was treated with a saturated NH_4_Cl solution. The ether layer was separated and dried over Na_2_SO_4_. The solvent was then evaporated from the solution *in vacuo* and the residue recrystallized from ethanol. The resulting solid, tris(4-bromophenyl)antimony, was dissolved in petroleum ether at 6 °C and then chlorine was passed slowly into the solution to yield the title compound as a white powder which was recrystallised from dichloromethane (Yield 43%). Anal. Calcd (%) for C_18_H_12_Cl_2_Br_3_Sb (Mr = 660.67): C, 32.72; H, 1.83; Cl, 10.73; Br, 36.28. Found (%): C, 32.66; H, 1.78; Cl, 10.81; Br, 36.23.

### Refinement

The C–H H atoms were positioned with idealized geometry and were refined isotropically with *U*_iso_(H) = 1.2 *U*_eq_(C).

### Crystal data

**Table:**

[SbCl_2_(C_6_H_4_Br)_3_]	*F*(000) = 2480
*M**_r_* = 660.66	*D*_x_ = 2.151 Mg m^−^^3^
Monoclinic, *C*2/*c*	Mo *K*α radiation, λ = 0.71073 Å
Hall symbol: -C 2yc	Cell parameters from 2728 reflections
*a* = 15.1050 (13) Å	θ = 2.9–26.1°
*b* = 20.124 (2) Å	µ = 7.49 mm^−^^1^
*c* = 15.1701 (14) Å	*T* = 298 K
β = 117.748 (1)°	Block, colourless
*V* = 4081.0 (7) Å^3^	0.26 × 0.22 × 0.12 mm
*Z* = 8	

### Data collection

**Table:**

Bruker SMART CCD area-detector diffractometer	3582 independent reflections
Radiation source: fine-focus sealed tube	2159 reflections with *I* > 2σ(*I*)
Graphite monochromator	*R*_int_ = 0.060
φ and ω scans	θ_max_ = 25.0°, θ_min_ = 2.5°
Absorption correction: multi-scan (*SADABS*; Sheldrick, 1996)	*h* = −17→16
*T*_min_ = 0.246, *T*_max_ = 0.467	*k* = −23→15
10608 measured reflections	*l* = −18→17

### Refinement

**Table:**

Refinement on *F*^2^	Primary atom site location: structure-invariant direct methods
Least-squares matrix: full	Secondary atom site location: difference Fourier map
*R*[*F*^2^ > 2σ(*F*^2^)] = 0.040	Hydrogen site location: inferred from neighbouring sites
*wR*(*F*^2^) = 0.097	H-atom parameters constrained
*S* = 0.91	*w* = 1/[σ^2^(*F*_o_^2^) + (0.0456*P*)^2^] where *P* = (*F*_o_^2^ + 2*F*_c_^2^)/3
3582 reflections	(Δ/σ)_max_ < 0.001
217 parameters	Δρ_max_ = 0.90 e Å^−^^3^
0 restraints	Δρ_min_ = −0.61 e Å^−^^3^

### Fractional atomic coordinates and isotropic or equivalent isotropic displacement parameters (Å^2^)

**Table:**

	*x*	*y*	*z*	*U*_iso_*/*U*_eq_	
Sb1	0.47083 (3)	0.31132 (2)	0.45721 (3)	0.05088 (16)	
C1	0.3297 (5)	0.3457 (3)	0.3455 (5)	0.0502 (16)	
C2	0.3204 (5)	0.3658 (4)	0.2544 (5)	0.065 (2)	
H2	0.3751	0.3632	0.2420	0.078*	
C3	0.2300 (5)	0.3899 (4)	0.1816 (5)	0.069 (2)	
H3	0.2225	0.4027	0.1196	0.083*	
C4	0.1522 (5)	0.3943 (3)	0.2032 (5)	0.0574 (18)	
C5	0.1589 (5)	0.3734 (4)	0.2912 (6)	0.0641 (19)	
H5	0.1039	0.3764	0.3030	0.077*	
C6	0.2484 (5)	0.3475 (3)	0.3632 (5)	0.0580 (18)	
H6	0.2535	0.3316	0.4229	0.070*	
C7	0.5462 (5)	0.3531 (3)	0.6018 (5)	0.0496 (16)	
C8	0.6001 (5)	0.3122 (3)	0.6817 (5)	0.0620 (18)	
H8	0.6003	0.2666	0.6719	0.074*	
C9	0.6535 (5)	0.3378 (4)	0.7757 (5)	0.066 (2)	
H9	0.6909	0.3101	0.8293	0.079*	
C10	0.6509 (5)	0.4053 (4)	0.7895 (5)	0.0617 (19)	
C11	0.5935 (5)	0.4463 (4)	0.7094 (5)	0.070 (2)	
H11	0.5900	0.4916	0.7194	0.084*	
C12	0.5421 (5)	0.4199 (4)	0.6156 (5)	0.0609 (19)	
H12	0.5046	0.4473	0.5616	0.073*	
C13	0.5404 (5)	0.2323 (3)	0.4187 (4)	0.0532 (17)	
C14	0.6240 (6)	0.2423 (4)	0.4084 (6)	0.079 (2)	
H14	0.6518	0.2845	0.4169	0.095*	
C15	0.6669 (6)	0.1895 (5)	0.3854 (7)	0.096 (3)	
H15	0.7243	0.1963	0.3786	0.115*	
C16	0.6274 (6)	0.1278 (4)	0.3725 (5)	0.067 (2)	
C17	0.5454 (6)	0.1176 (4)	0.3810 (6)	0.080 (2)	
H17	0.5189	0.0750	0.3726	0.096*	
C18	0.4980 (6)	0.1699 (4)	0.4024 (6)	0.072 (2)	
H18	0.4388	0.1628	0.4056	0.087*	
Cl1	0.38762 (13)	0.23427 (9)	0.52062 (13)	0.0672 (5)	
Cl2	0.55520 (13)	0.39558 (9)	0.40399 (13)	0.0642 (5)	
Br1	0.03004 (6)	0.43361 (5)	0.10847 (6)	0.0882 (3)	
Br2	0.72426 (7)	0.44321 (5)	0.91767 (6)	0.0951 (3)	
Br3	0.68965 (7)	0.05538 (5)	0.34275 (8)	0.1048 (4)	

### Atomic displacement parameters (Å^2^)

**Table:**

	*U*^11^	*U*^22^	*U*^33^	*U*^12^	*U*^13^	*U*^23^
Sb1	0.0547 (3)	0.0527 (3)	0.0488 (3)	−0.0014 (3)	0.0270 (2)	0.0039 (2)
C1	0.052 (4)	0.049 (4)	0.051 (4)	0.001 (3)	0.025 (3)	0.003 (3)
C2	0.064 (5)	0.081 (5)	0.052 (4)	0.000 (4)	0.029 (4)	0.009 (4)
C3	0.073 (5)	0.082 (6)	0.051 (4)	−0.007 (5)	0.029 (4)	0.011 (4)
C4	0.056 (4)	0.042 (4)	0.065 (5)	−0.004 (4)	0.021 (4)	−0.009 (4)
C5	0.057 (5)	0.067 (5)	0.073 (5)	0.007 (4)	0.034 (4)	−0.003 (4)
C6	0.067 (5)	0.066 (5)	0.054 (4)	0.007 (4)	0.039 (4)	0.007 (4)
C7	0.055 (4)	0.053 (4)	0.046 (4)	0.002 (4)	0.028 (3)	0.008 (3)
C8	0.072 (5)	0.045 (4)	0.063 (5)	0.001 (4)	0.027 (4)	0.003 (4)
C9	0.078 (5)	0.060 (5)	0.049 (5)	0.005 (4)	0.019 (4)	0.013 (4)
C10	0.064 (5)	0.065 (5)	0.056 (4)	0.002 (4)	0.028 (4)	0.005 (4)
C11	0.084 (5)	0.062 (5)	0.055 (5)	0.008 (4)	0.025 (4)	−0.001 (4)
C12	0.067 (5)	0.056 (5)	0.050 (4)	0.012 (4)	0.019 (4)	0.013 (4)
C13	0.058 (4)	0.059 (5)	0.041 (4)	−0.006 (4)	0.022 (3)	0.001 (3)
C14	0.080 (6)	0.067 (5)	0.112 (7)	−0.016 (5)	0.062 (5)	−0.017 (5)
C15	0.079 (6)	0.094 (7)	0.138 (8)	−0.013 (6)	0.071 (6)	−0.018 (7)
C16	0.063 (5)	0.077 (6)	0.058 (5)	0.010 (5)	0.025 (4)	−0.006 (4)
C17	0.088 (6)	0.068 (6)	0.087 (6)	−0.011 (5)	0.044 (5)	−0.016 (5)
C18	0.077 (5)	0.067 (5)	0.085 (6)	−0.007 (5)	0.048 (5)	−0.008 (4)
Cl1	0.0733 (12)	0.0662 (12)	0.0739 (13)	−0.0075 (10)	0.0442 (10)	0.0113 (10)
Cl2	0.0704 (12)	0.0661 (12)	0.0652 (11)	−0.0121 (10)	0.0390 (10)	0.0037 (10)
Br1	0.0780 (6)	0.0830 (6)	0.0792 (6)	0.0122 (5)	0.0160 (5)	0.0039 (5)
Br2	0.1167 (7)	0.0916 (7)	0.0552 (5)	−0.0110 (6)	0.0218 (5)	−0.0049 (5)
Br3	0.1026 (7)	0.0967 (7)	0.1100 (8)	0.0208 (6)	0.0453 (6)	−0.0252 (6)

### Geometric parameters (Å, º)

**Table:**

Sb1—C1	2.129 (6)	C8—H8	0.9300
Sb1—C7	2.119 (6)	C9—C10	1.377 (10)
Sb1—C13	2.132 (7)	C9—H9	0.9300
Sb1—Cl1	2.4566 (16)	C10—C11	1.388 (9)
Sb1—Cl2	2.4716 (17)	C10—Br2	1.893 (7)
C1—C6	1.373 (8)	C11—C12	1.372 (9)
C1—C2	1.384 (8)	C11—H11	0.9300
C2—C3	1.385 (9)	C12—H12	0.9300
C2—H2	0.9300	C13—C14	1.357 (8)
C3—C4	1.363 (9)	C13—C18	1.380 (9)
C3—H3	0.9300	C14—C15	1.371 (10)
C4—C5	1.357 (9)	C14—H14	0.9300
C4—Br1	1.904 (7)	C15—C16	1.351 (10)
C5—C6	1.384 (9)	C15—H15	0.9300
C5—H5	0.9300	C16—C17	1.320 (9)
C6—H6	0.9300	C16—Br3	1.900 (7)
C7—C12	1.366 (9)	C17—C18	1.392 (10)
C7—C8	1.375 (9)	C17—H17	0.9300
C8—C9	1.370 (9)	C18—H18	0.9300
			
C7—Sb1—C1	123.5 (2)	C9—C8—H8	119.6
C7—Sb1—C13	118.9 (2)	C7—C8—H8	119.6
C1—Sb1—C13	117.6 (2)	C8—C9—C10	118.9 (7)
C7—Sb1—Cl1	88.37 (17)	C8—C9—H9	120.5
C1—Sb1—Cl1	90.64 (18)	C10—C9—H9	120.5
C13—Sb1—Cl1	92.52 (19)	C9—C10—C11	120.2 (7)
C7—Sb1—Cl2	87.45 (17)	C9—C10—Br2	120.6 (6)
C1—Sb1—Cl2	89.71 (17)	C11—C10—Br2	119.2 (6)
C13—Sb1—Cl2	91.56 (19)	C12—C11—C10	119.9 (7)
Cl1—Sb1—Cl2	175.20 (6)	C12—C11—H11	120.0
C6—C1—C2	120.3 (6)	C10—C11—H11	120.0
C6—C1—Sb1	120.6 (5)	C7—C12—C11	119.7 (6)
C2—C1—Sb1	119.2 (5)	C7—C12—H12	120.1
C1—C2—C3	120.1 (6)	C11—C12—H12	120.1
C1—C2—H2	119.9	C14—C13—C18	119.5 (7)
C3—C2—H2	119.9	C14—C13—Sb1	121.7 (6)
C4—C3—C2	118.2 (6)	C18—C13—Sb1	118.8 (5)
C4—C3—H3	120.9	C13—C14—C15	119.3 (7)
C2—C3—H3	120.9	C13—C14—H14	120.3
C5—C4—C3	122.6 (6)	C15—C14—H14	120.3
C5—C4—Br1	118.3 (5)	C16—C15—C14	121.3 (7)
C3—C4—Br1	119.1 (6)	C16—C15—H15	119.4
C4—C5—C6	119.4 (6)	C14—C15—H15	119.4
C4—C5—H5	120.3	C17—C16—C15	120.0 (8)
C6—C5—H5	120.3	C17—C16—Br3	119.7 (7)
C1—C6—C5	119.3 (6)	C15—C16—Br3	120.4 (7)
C1—C6—H6	120.4	C16—C17—C18	120.9 (8)
C5—C6—H6	120.4	C16—C17—H17	119.6
C12—C7—C8	120.3 (6)	C18—C17—H17	119.6
C12—C7—Sb1	120.5 (5)	C13—C18—C17	118.9 (7)
C8—C7—Sb1	119.2 (5)	C13—C18—H18	120.5
C9—C8—C7	120.8 (7)	C17—C18—H18	120.5

### Hydrogen-bond geometry (Å, º)

**Table:**

*D*—H···*A*	*D*—H	H···*A*	*D*···*A*	*D*—H···*A*
C2—H2···Cl2^i^	0.93	2.93	3.723 (7)	144
C17—H17···Br1^ii^	0.93	2.99	3.900 (9)	167

Symmetry codes: (i) −*x*+1, *y*, −*z*+1/2; (ii) −*x*+1/2, *y*−1/2, −*z*+1/2.
